# Kinetic analysis of the translocator protein positron emission tomography ligand [^18^F]GE-180 in the human brain

**DOI:** 10.1007/s00259-016-3444-z

**Published:** 2016-06-28

**Authors:** Claire Feeney, Gregory Scott, Joel Raffel, S. Roberts, Christopher Coello, Amy Jolly, Graham Searle, A. P. Goldstone, David J. Brooks, Richard S. Nicholas, William Trigg, Roger N. Gunn, David J. Sharp

**Affiliations:** 1 Division of Brain Sciences, Hammersmith Hospital Campus, Imperial College London, London, UK; 2 Institute of Clinical Medicine, Aarhus University, Aarhus, Denmark; 3 GE Healthcare Ltd, Amersham, UK; 4 Computational, Cognitive and Clinical Neuroimaging Laboratory, Hammersmith Hospital, 3rd Floor, Burlington Danes Building, Du Cane Road, London, W12 0NN UK

**Keywords:** Positron emission tomography (PET), GE180, Translocator protein (TSPO), Kinetic analysis, Quantification, Neuroinflammation

## Abstract

**Purpose:**

PET can image neuroinflammation by targeting the translocator protein (TSPO), which is upregulated in activated microglia. The high nonspecific binding of the first-generation TSPO radioligand [^11^C]PK-11195 limits accurate quantification. [^18^F]GE-180, a novel TSPO ligand, displays superior binding to [^11^C]PK-11195 in vitro. Our objectives were to: (1) evaluate tracer characteristics of [^18^F]GE-180 in the brains of healthy human subjects; and (2) investigate whether the TSPO Ala147Thr polymorphism influences outcome measures.

**Methods:**

Ten volunteers (five high-affinity binders, HABs, and five mixed-affinity binders, MABs) underwent a dynamic PET scan with arterial sampling after injection of [^18^F]GE-180. Kinetic modelling of time–activity curves with one-tissue and two-tissue compartment models and Logan graphical analysis was applied to the data. The primary outcome measure was the total volume of distribution (*V*_T_) across various regions of interest (ROIs). Secondary outcome measures were the standardized uptake values (SUV), the distribution volume and SUV ratios estimated using a pseudoreference region.

**Results:**

The two-tissue compartment model was the best model. The average regional delivery rate constant (*K*_1_) was 0.01 mL cm^−3^ min^−1^ indicating low extraction across the blood–brain barrier (1 %). The estimated median *V*_T_ across all ROIs was also low, ranging from 0.16 mL cm^−3^ in the striatum to 0.38 mL cm^−3^ in the thalamus. There were no significant differences in *V*_T_ between HABs and MABs across all ROIs.

**Conclusion:**

A reversible two-tissue compartment model fitted the data well and determined that the tracer has a low first-pass extraction (approximately 1 %) and low *V*_T_ estimates in healthy individuals. There was no observable dependency on the rs6971 polymorphism as compared to other second-generation TSPO PET tracers. Investigation of [^18^F]GE-180 in populations with neuroinflammatory disease is needed to determine its suitability for quantitative assessment of TSPO expression.

**Electronic supplementary material:**

The online version of this article (doi:10.1007/s00259-016-3444-z) contains supplementary material, which is available to authorized users.

## Introduction

The translocator protein (TSPO) is a mitochondrial transporter involved in varied intracellular processes, but its expression in the central nervous system (CNS) is relatively low under normal physiological conditions [1]. However, activation of microglial cells caused by inflammatory stimuli results in significant upregulation of TSPO expression [2]. TSPO quantification with PET provides a measure of intrinsic neuroinflammation in a variety of CNS diseases. Early PET studies used the isoquinoline [^11^C]PK-11195 to measure TSPO binding and detected elevations across a range of conditions including multiple sclerosis [3], Huntington’s disease [4], Alzheimer’s disease [5, 6], traumatic brain injury [7] and ischaemic stroke [8]. However, the use of [^11^C]PK-11195 is limited by a high nonspecific signal, making nonstandard approaches to data analysis necessary [9]. In addition, because ^11^C has a half-life of 20.3 min, the use of [^11^C]PK-11195 is restricted to locations with an on-site cyclotron.

A number of second-generation TSPO ligands have been developed recently with the promise of improved signal-to-noise ratio and greater specific binding. [^18^F]GE-180 is a novel fluorinated radiotracer that binds to the TSPO with high affinity [10]. Developed from a series of tricyclic indoles, [^18^F]GE-180 has demonstrated superior specific binding affinity to [^11^C]PK-11195 in animal models of acute neuroinflammation [11] and stroke [12]. The ^18^F radiolabel, with a half-life of 109.8 min, also makes [^18^F]GE-180 more suitable than ^11^C-based compounds for long-distance distribution, enabling widespread clinical use.

Other second-generation TSPO radiotracers (e.g. [^11^C]PBR-28, [^18^F]PBR-06, [^11^C]-DAA1106, [^11^C]-DPA713, [^18^F] FEPPA) show binding affinities influenced by a TSPO polymorphism expressed by individuals and have been classified as high-affinity binders (HABs), mixed-affinity binders (MABs) and low-affinity binders (LABs) [13]. Expression of the TSPO Ala147Thr polymorphism results in MAB or LAB depending on whether one or two copies are present [14]. Here, we report a study in healthy subjects using [^18^F]GE-180 PET imaging. The primary aim was to investigate tracer kinetics and quantification in healthy human subjects. The secondary aim was to investigate whether there were differences in binding between HABs and MABs.

## Materials and methods

### Human subjects

This study was approved by the Westminster Research Ethics Committee, London (13/LO/1596), the Riverside Research Ethics Committee (13/LO/1916), and the Administration of Radioactive Substances Advisory Committee (no. 631/336/30788). Research was conducted in accordance with the principles of the Declaration of Helsinki [15]. All subjects gave written, informed consent.

Ten healthy volunteers (seven men), mean age 41 ± 9 years (range 28 – 56 years), mean weight 81.8 ± 13 kg, were included in the study. A screening assessment was carried out that included full medical and drug history, blood pressure, height, weight, Allen’s test for patency of the ulnar anastomosis, and the Structured Clinical Interview for DSM disorders (SCID). Blood samples were taken for analysis of full blood count, renal profile, clotting screen and TSPO genotyping. Exclusion criteria included pregnancy, a history of prior or current psychiatric or neurological disease, abuse of alcohol or drugs and contraindication to arterial line placement.

### TSPO genotyping

DNA was extracted using a Qiagen QIAmp DNA blood mini kit. TSPO genotyping of the c.439A > G (p.Thr147A1a) (SNP rs6971) was performed using a TaqMan allelic discrimination assay. LABs (one subject) were excluded from the imaging component of the study. During the study design it was felt that on ethical and economical grounds LABs should not be exposed to this novel tracer and instead that the focus should be on MABs and HABs. Of the ten subjects eligible for imaging, five were HABs and five were MABs.

### Synthesis of [^18^F]GE-180

[^18^F]-Fluorine was produced by the ^18^O(p,n)^18^F nuclear reaction on a GE PETtrace 8 cyclotron (The Grove Centre, Amersham, UK). All radiochemistry was performed on a GE FASTlab synthesizer with single-use cassettes. The average synthesis time was 43 mins, radiochemical yield was 43 % and purity was greater than 95 % [16]. The radiotracer was manufactured by GE Healthcare (The Grove Centre, Amersham, UK), transported to Hammersmith Hospital, London, and used within 8 h of manufacture.

### Positron emission tomography scanning and image reconstruction

All subjects were scanned at the Clinical Imaging Facility, Imperial College London, Hammersmith Hospital. Prior to PET scanning, an arterial cannula was inserted under local anaesthesia (2 % lidocaine) into the radial artery to allow arterial blood sampling. An antecubital venous cannula was inserted for radiotracer administration.

PET studies were performed on a Biograph 6 (six-slice CT) scanner after administration of 182 ± 3.1 MBq via intravenous bolus injection over 10 s followed by a 10-mL saline flush. A low-dose CT scan preceded the PET acquisition to allow correction for tissue attenuation. Emission data were then acquired over 90 min in list mode and reconstructed as 24 temporal frames (6 × 15, 3 × 60, 5 × 120, 5 × 300, 5 × 600 s) using filtered back-projection (matrix size 168 × 168, zoom 2.6, 5-mm gaussian filter, pixel size 1.56 × 1.56, slice thickness 3 mm) with and without attenuation correction. Standard corrections for scatter, decay and random counts were applied.

### Whole-blood, plasma activity and parent fraction of [^18^F]GE-180

Arterial blood activity was measured every second for the first 15 min of the 90-min scan using an automated blood sampling system (ABSS Allog, Mariefred, Sweden) connected to the subject via a 1.5 m × 1.0 mm diameter polytetrafluoroethylene line (blood withdrawal rate 2.5 ml/min). In addition, blood samples (10 mL) were collected manually from the radial artery at 0, 5, 10, 15, 30, 50, 70 and 90 min to assay whole-blood and plasma activity. Plasma was obtained by centrifugation for 3 min at 1,800 *g*. Activity in whole blood and plasma was measured in a CAPRAC-t well counter over 10 – 60 s. The first 15 min of continuous whole-blood activity was combined with the activity from discrete samples to generate the whole-blood activity curve for use in data modelling. The continuous plasma-to-blood ratio was estimated using a constant model and a total plasma activity curve was obtained by correcting the whole-blood curve for this partition between plasma and blood.

The parent fraction of [^18^F]GE-180 was measured by high-performance liquid chromatography (Agilent 1,100 and 1,260 series) of discrete plasma samples (3.5 mL). The parent fraction was fitted to a single exponential plus a constant model. The total plasma activity was multiplied by this parent fraction and then smoothed post peak by fitting to a triexponential function to generate an arterial parent plasma input function. A delay correction of up to +30 s was applied to the input function prior to fitting the kinetic modelling. This was performed to account for delay in the 1.5-m tube (28.3 s) and delay between the radial artery and the brain.

### Magnetic resonance imaging

To provide additional anatomical information to aid analysis of the PET data, each subject underwent a structural T1-weighted MRI scan on a Siemens Verio 3-T scanner (matrix size 256 × 256, voxel size 1 × 1 × 1 mm , TR 9.63 ms, TE 4.74 ms, flip angle 9°).

### Data analysis

**Fig. 1:**
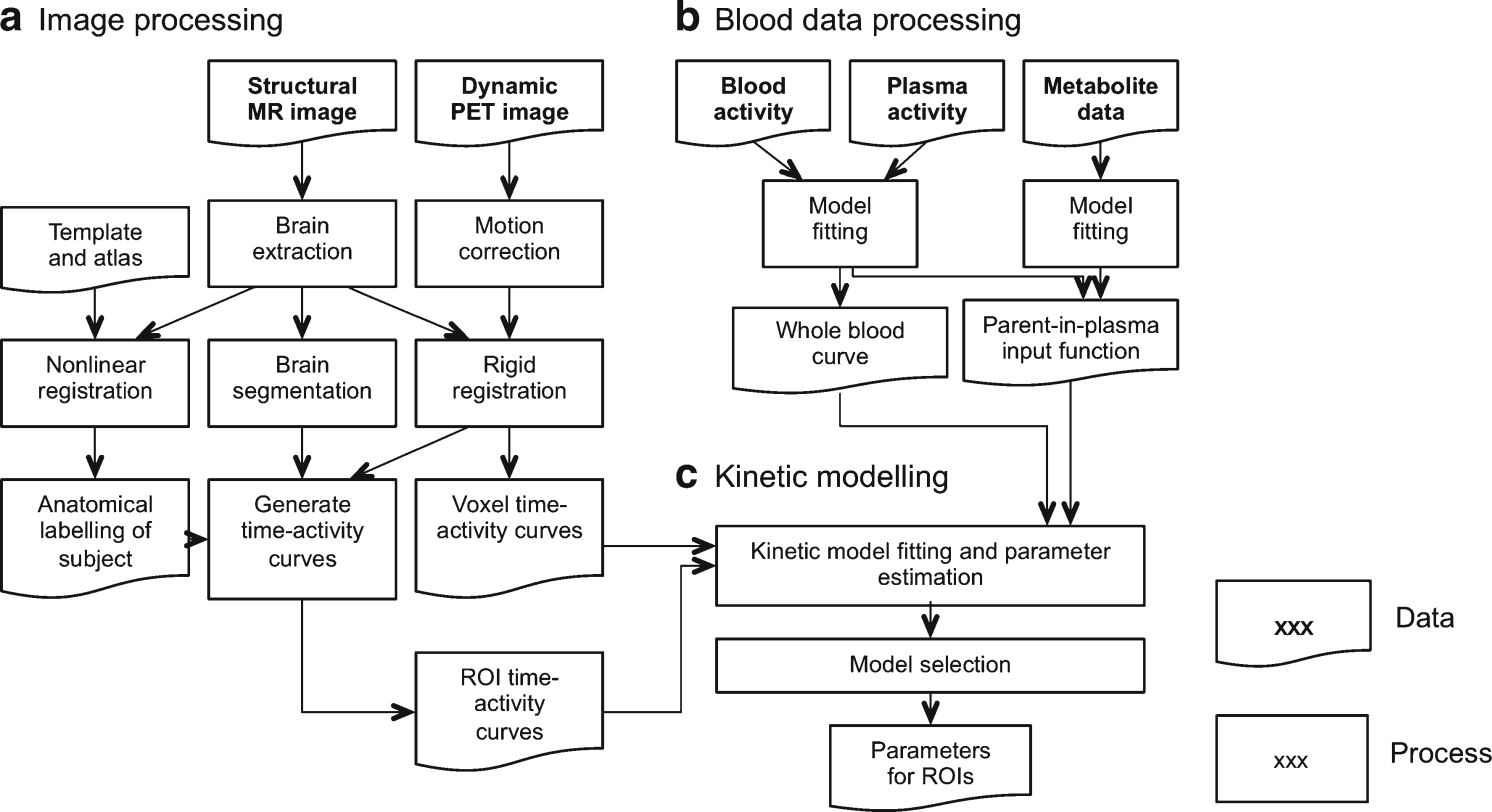
Overview of data analysis. The MIAKAT™ analysis toolkit was used for image processing (**a**), blood data processing (**b**) and kinetic modelling (**c**) of the PET data

A high level overview of the data analysis is provided in Fig. 1. We used the PET data analysis and kinetic modelling toolkit, MIAKAT™ (www.miakat.org), which incorporates software from SPM5 (Wellcome Trust Centre for Neuroimaging) and FSL (FMRIB, University of Oxford) [17]. The brain was initially extracted from the T1-weighted MR images. The CIC neuroanatomical atlas [18] was nonlinearly registered to the individual’s extracted brain in order to generate a personalized set of anatomically parcellated regions [18]. Frame-by-frame motion correction of the dynamic (without attenuation correction) PET data was performed using mutual information coregistration of the individual frames to a reference frame. An average motion-corrected PET image was generated and used for coregistration with the T1-weighted MR image. Finally, regional tissue time–activity curves (TACs) were generated for each region of interest (ROI) defined from the CIC atlas that had been transformed into each individual’s image space.

Datasets were analysed with a one-tissue compartment (1TC) model and a two-tissue compartment (2TC) model and the Logan graphical method, using the metabolite-corrected plasma input function as previously described [19] with a fixed 5 % blood volume correction. The primary outcome measures were the radioligand delivery rate constant (*K*_1_; mL cm^−3^ min^−1^), total distribution volume (*V*_T_: mL cm^−3^), and standardized uptake values (SUV). The SUV ratio (SUVR) and distribution volume ratio (DVR) were estimated using the cortical grey matter as a pseudo reference region. This region was chosen as there is no true reference region for TSPO, but the cortical grey matter has been used as a reference in previous work as healthy brain usually shows low TSPO expression in this region [20].

### Time stability analysis

To investigate the stability of *V*_T_ over different scan durations, a time stability analysis was performed by analysing data for total time windows that ranged between 40 and 90 min in 10-min increments.

### Statistical analysis

The Akaike information criterion (AIC) was used to select the most appropriate compartment model [21], where lower AIC was indicative of a more parsimonious model. To compare characteristics between the genetic groups (HAB/MAB), Fisher’s exact test (gender) and the Mann Whitney *U* test (age, weight, injected dose) were used. To evaluate differences in TACs for blood data between the genetic groups, a repeated measures analysis of variance (ANOVA) was used with time as the within-subjects factor and genotype as the between-subjects factor. A repeated measures ANOVA was also used to compare outcome measures across multiple ROIs, where ROI was used as the within-subjects factor.

## Results

Injection of [^18^F]GE-180 caused no pharmacological effects based on patient reports, blood pressure, pulse, respiration rate and oxygen saturation after radioligand administration. There were no significant differences in gender, age or weight between HABs and MABs. With regard to correlations between age and the principal outcome measure, there were no significant correlations between age and *V*_T_ in either HABs and MABs in any of the ROIs studied (Spearman’s rho = −0.3 – 0.7 *p* = 0.188 – 0.873 in HABs; Spearman’s rho = 0.1 – 0.8, *p* = 0.104 – 0.94 in MABs). There were also no significant relationships between age and outcome measures when added as a covariate in the repeated measures ANOVA. Demographic data are provided in Supplementary Table 1.

### Plasma data

**Fig. 2:**
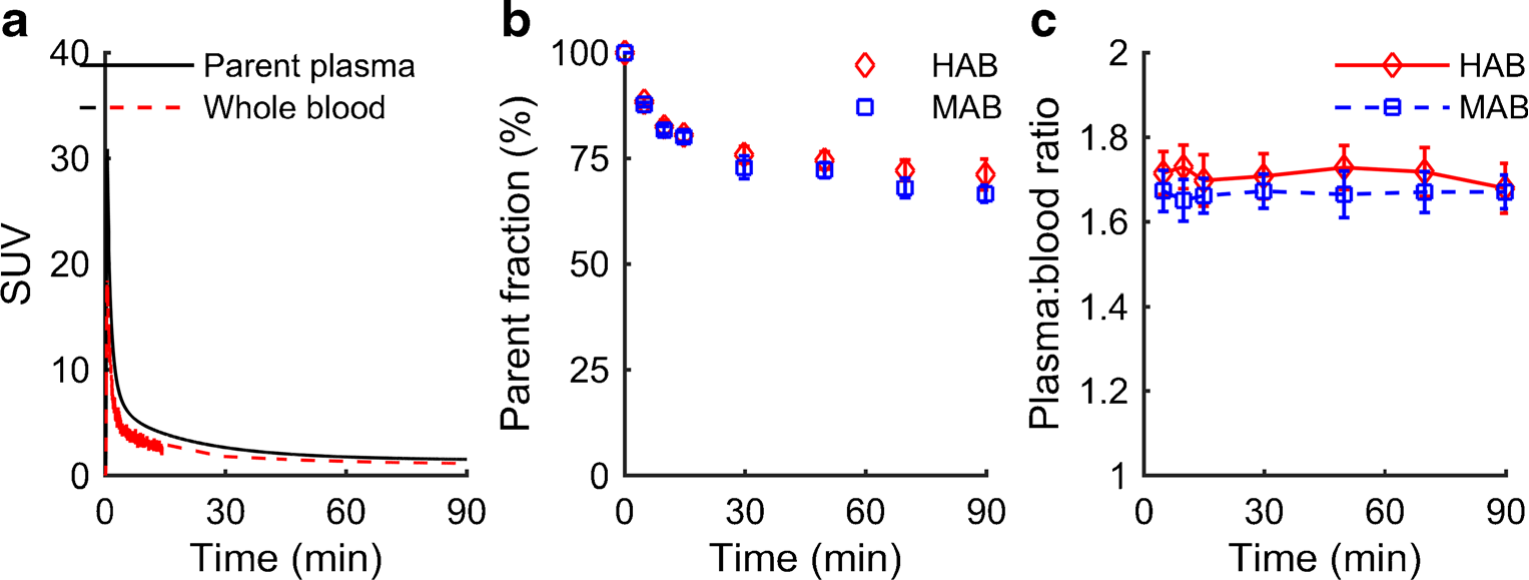
Blood data. **a** Whole blood (*red*) and parent in plasma (*black*) curves for one subject. **b** The fraction of unchanged parent compound over time for high-affinity binders (*HAB*, *red*) and mixed-affinity binders (*MAB*, *blue*). **c** Plasma-to-blood ratio over time for HABs and MABs. Values are plotted in **b** and **c** as means ± standard error of the mean

In plasma, the concentration of [^18^F]GE-180 peaked at about 45 s and then showed a rapid decrease (Fig. 2a). The fraction of unchanged [^18^F]GE-180 over time is shown in Fig. 2b. The parent compound accounted for 65.0 – 81.7 % (range across subjects) of the total concentration in plasma at 30 min, and 57.3 – 78.3 % at 90 min. Three polar radioactive metabolites were identified over the course of the 90 min scanning window (<10 % of parent compound). The plasma-to-blood ratio remained constant at about 1.69 (Fig. 2c) across subjects. There were no significant differences in profiles of plasma over blood (F(1,48) = 0.407, *p* = 0.541), parent fraction (F(1,48) = 0.871, *p* = 0.378) or parent in plasma between genetic groups (F(1,48) = 0.130, *p* = 0.728). There was no interaction of genotype with time for any of these profiles (*p* > 0.204). The parent in plasma profiles for HABs and MABs are shown in Supplementary Fig. 1.

### Tissue data

**Fig. 3:**
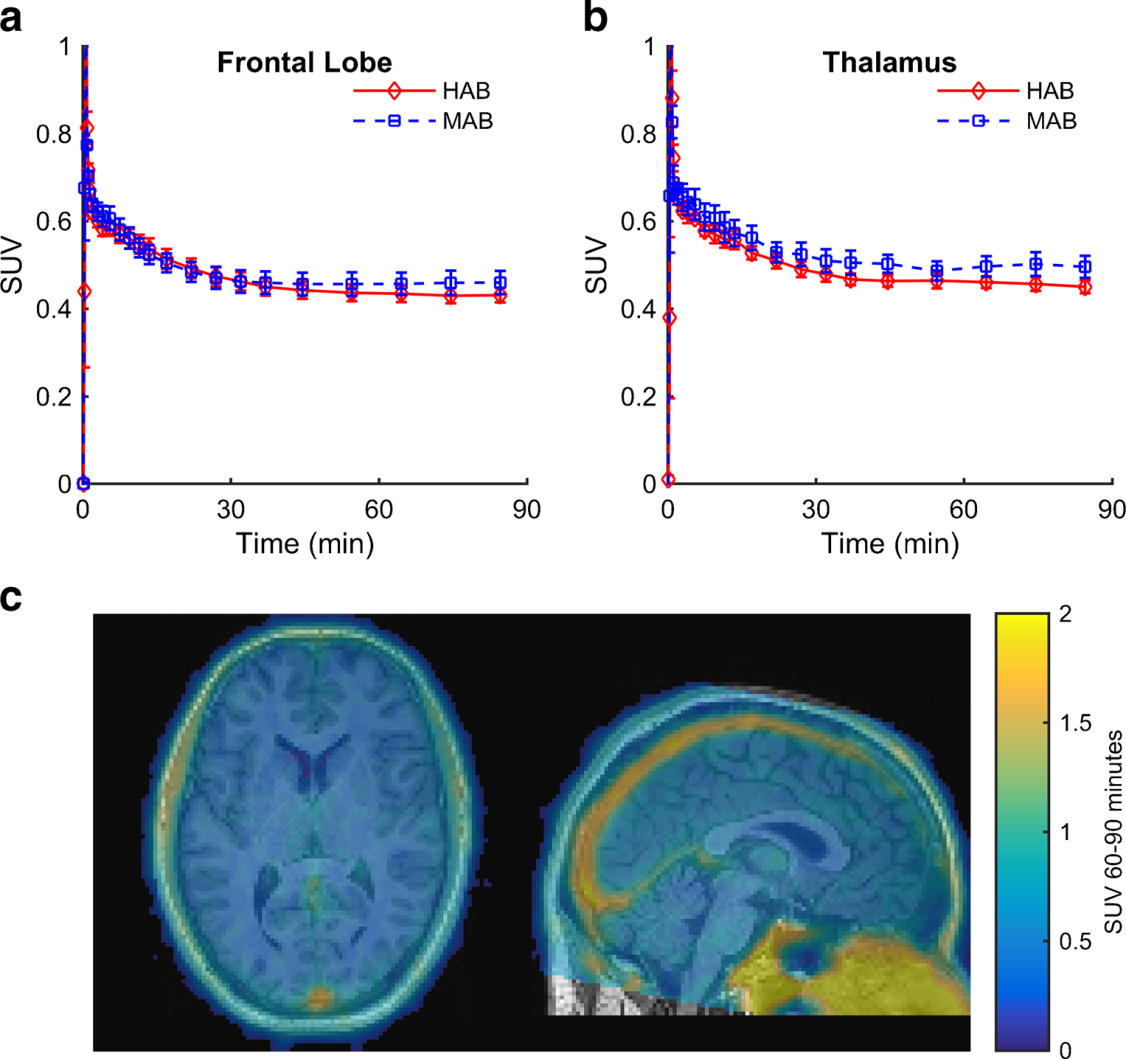
Imaging data. Time–activity curves (TACs) are shown for high-affinity binders (*HAB*) and mixed-affinity binders (*MAB*) in the frontal grey matter (**a**) and thalamus (**b**). Standardized uptake values (SUV) are plotted as mean ± standard error of the mean. **c** SUV image calculated from the 60 – 90-min PET frames superimposed on the coregistered T1-weighted MR image in a representative MAB subject

The concentration of the ligand in the brain peaked at around 1 min and then showed rapid washout in all subjects. Group-averaged tissue TACs for the frontal grey matter and thalamus are shown in Fig. 3a, b. A 60 – 90-min SUV image in a representative MAB subject is shown in Fig. 3c. Overall, there was low uptake of the tracer in brain with images being dominated by signal from blood vessels. There were no significant differences in SUV curves between genetic groups (F(1,48) = 1.396, *p* = 0.271). There was no interaction of genotype with time (*P* = 0.684).

### Kinetic analysis

**Fig. 4:**
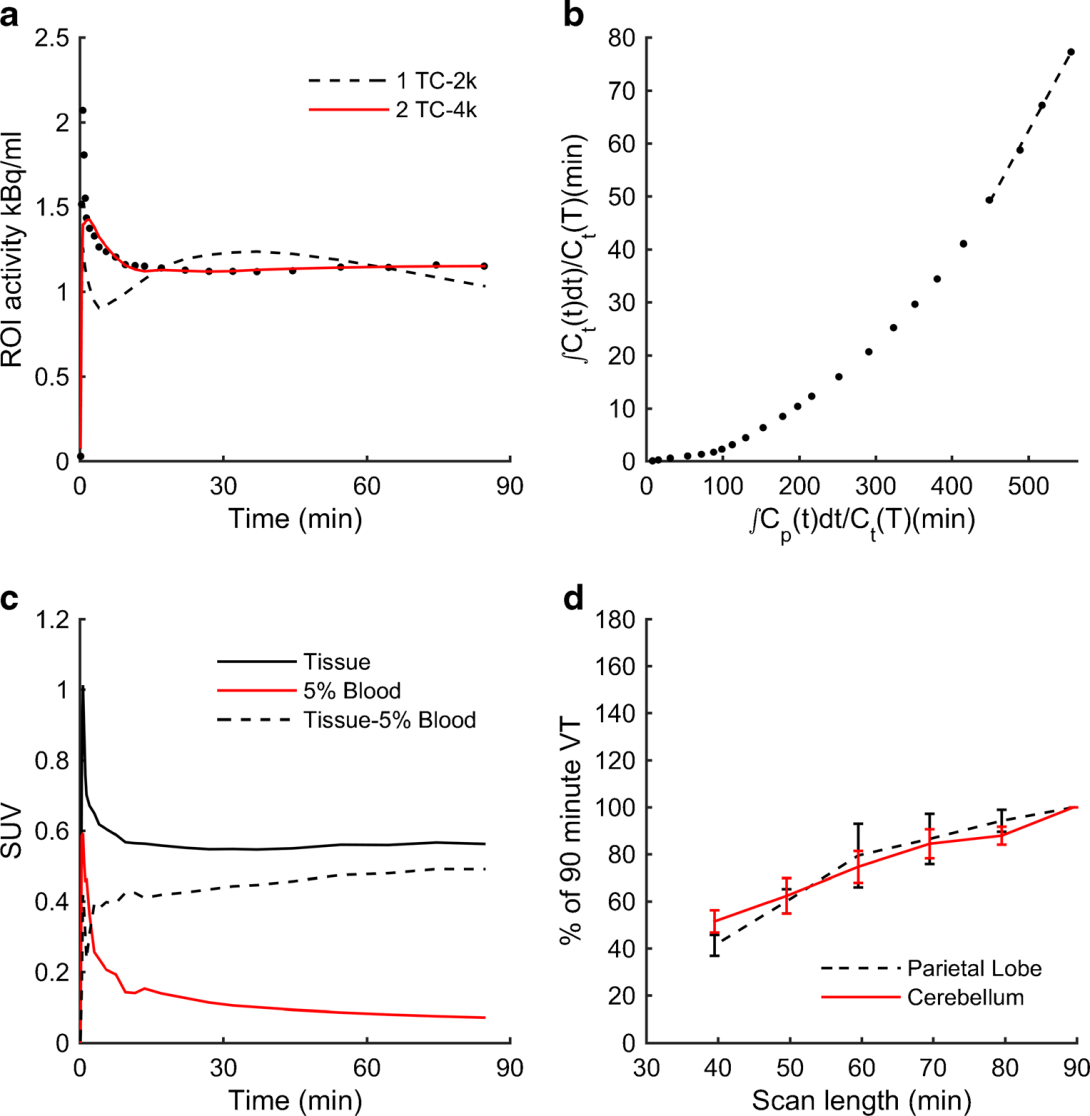
Kinetic modelling and time stability analysis. **a** Model fits are shown for one-tissue compartment model (reversible 1TC, *dashed line*) and two-tissue compartment model (reversible 2TC, *red line*) against a time–activity curve (*black dots*) for the parietal lobe. **b** Logan plot (*black dashed line* the most linear portion of the curve) for the same time–activity curve as in **a**. **c** Parietal lobe time–activity curve (*solid line*), 5 % whole-blood activity curve (*red line*) and the curve of the difference between the two curves (*dashed line*) highlights the significant contribution of blood signal to the time–activity curve. **d** Estimates of two-tissue compartment model *V*_T_ calculated for 40 – 90-min scan windows in 10-min increments plotted as the absolute percentage difference compared to the final 90-min *V*_T_ for the parietal lobe and cerebellum grey matter regions of interest. The values plotted are means ± standard error of the mean

The results of the kinetic modelling are shown in Table 1. The 2TC model was superior to the 1TC model as judged by a lower AIC in all ROIs except the striatum. Example model fits for the 1TC and 2TC models are shown in Fig. 4a in a representative MAB subject. The first 10 min of Fig. 4a and Fig. 4c are shown in more detail in Supplementary Fig. 2. The 2TC model generally showed a good fit to the data except for an initial small mismatch in the blood volume peak, which was considered not to have affected *V*_T_ estimates. When we included the 2TC-fit model, it did not outperform the 2TC-fix based on the AIC. Blood volume estimates ranged from 6.3 % to 10.5 % (mean 8.4 %) across all ROIs. Therefore, the 2TC-fix was selected as the model to use for further analysis and gave average rate constants (across all regions and subjects) of *K*_1_ = 0.013 mL cm^−3^ min^−1^, *k*_2_ = 0.229 min^−1^, *k*_3_ = 0.035 min^−1^ and *k*_4_ = 0.010 min^−1^, resulting in *V*_T_ = 0.311 mL cm^−3^ (Table 1).

**Table 1:** Parameter estimates and model fits

Model	Region	*K*_1_ (mL/min)	*k*_2_ (/min)	*k*_3_ (/min)	*k*_4_ (/min)	*V*_T_ (mL/cm^3^)	Distribution volume ratio	AIC wins^a^
1TC	Frontal lobe	0.00472 (0.0041 – 0.006)	0.0271 (0.023 – 0.028)			0.171 (0.15 – 0.22)	0.939 (0.94 – 0.95)	2/10
	Parietal lobe	0.00513 (0.004 – 0.0061)	0.027 (0.024 – 0.028)			0.19 (0.16 – 0.22)	0.969 (0.96 – 1)	1/10
	Temporal lobe	0.00514 (0.0048 – 0.006)	0.0266 (0.025 – 0.028)			0.182 (0.17 – 0.24)	1.04 (1 – 1)	0/10
	Occipital lobe	0.00621 (0.0057 – 0.0074)	0.03 (0.025 – 0.032)			0.214 (0.19 – 0.25)	1.12 (1.1 – 1.2)	0/10
	Thalamus	0.00555 (0.0046 – 0.0058)	0.0261 (0.024 – 0.028)			0.182 (0.17 – 0.24)	1.03 (0.96 – 1.1)	0/10
	Striatum	0.00358 (0.003 – 0.0046)	0.0241 (0.021 – 0.025)			0.155 (0.14 – 0.2)	0.826 (0.78 – 0.89)	8/10
	Cerebellum	0.00656 (0.0054 – 0.0076)	0.0339 (0.034 – 0.034)			0.178 (0.16 – 0.22)	0.968 (0.91 – 1)	0/10
2TC	Frontal lobe	0.0102 (0.0089 – 0.013)	0.195 (0.15 – 1.6)	0.0301 (0.024 – 0.15)	0.00653 (0.004 – 0.017)	0.346 (0.26 – 0.5)	0.984 (0.96 – 1.2)	8/10
	Parietal lobe	0.0116 (0.011 – 0.014)	0.192 (0.15 – 0.25)	0.0334 (0.025 – 0.039)	0.00873 (0.0045 – 0.014)	0.33 (0.31 – 0.42)	1.01 (0.98 – 1.1)	9/10
	Temporal lobe	0.0143 (0.012 – 0.032)	0.217 (0.16 – 1.3)	0.0348 (0.03 – 0.15)	0.00927 (0.0063 – 0.016)	0.306 (0.27 – 0.42)	0.958 (0.93 – 1)	10/10
	Occipital lobe	0.0238 (0.019 – 0.031)	0.385 (0.2 – 0.62)	0.0395 (0.036 – 0.054)	0.0101 (0.0062 – 0.014)	0.35 (0.3 – 0.52)	1.07 (0.97 – 1.1)	10/10
	Thalamus	0.0118 (0.011 – 0.014)	0.16 (0.14 – 0.2)	0.0315 (0.026 – 0.042)	0.00881 (0.0062 – 0.012)	0.376 (0.28 – 0.41)	0.922 (0.81 – 1.1)	10/10
	Striatum	0.00413 (0.0033 – 0.0088)	1.14 (0.66 – 1.7)	4.89 (0.62 – 9.1)	0.14 (0.027 – 0.21)	0.155 (0.14 – 0.22)	0.451 (0.41 – 0.71)	2/10
	Cerebellum	0.0224 (0.016 – 1.3)	0.319 (0.23 – 78)	0.0331 (0.029 – 0.091)	0.0113 (0.0084 – 0.018)	0.281 (0.25 – 0.33)	0.916 (0.69 – 0.98)	10/10
Logan	Frontal lobe					0.265 (0.23 – 0.33)	0.931 (0.92 – 0.97)	
	Parietal lobe					0.284 (0.23 – 0.35)	0.996 (0.93 – 1)	
	Temporal lobe					0.312 (0.24 – 0.36)	1.01 (0.95 – 1.1)	
	Occipital lobe					0.34 (0.3 – 0.39)	1.14 (1.1 – 1.2)	
	Thalamus					0.3 (0.26 – 0.31)	0.991 (0.98 – 1)	
	Striatum					0.25 (0.21 – 0.29)	0.844 (0.7 – 0.92)	
	Cerebellum					0.29 (0.24 – 0.3)	0.99 (0.91 – 1)	

Data are presented as medians (interquartile ranges)

^a^For the 1TC model versus the 2TC model, the data shown are the proportions of the ten subjects for whom the model was the more parsimonious (defined as having the lower AIC)

*K*_1_ was low across all ROIs in all subjects, indicating low extraction across the blood–brain barrier (BBB). This is consistent with the low tissue uptake observed in the images and the predominance of the vasculature structures. There was no significant effect of genetic group on any of the four rate constants. The Logan graphical method was also used to estimate the *V*_T_ in each ROI (Table 1). A representative plot is shown in Fig. 4b. Pooled *V*_T_ estimates from the 2TC model were positively correlated with *V*_T_ from the Logan method (Pearson’s *r* = 0.630, *p* < 0.0001; regression equation *V*_T_(Logan) = 0.3 × *V*_T_(2TC) + 0.19). The tissue TAC minus the whole-blood radioactivity curve demonstrated that approximately 20 % of the activity in a typical ROI came from blood (Fig. 4c).

### Time stability analysis and outcome measures

**Fig. 5:**
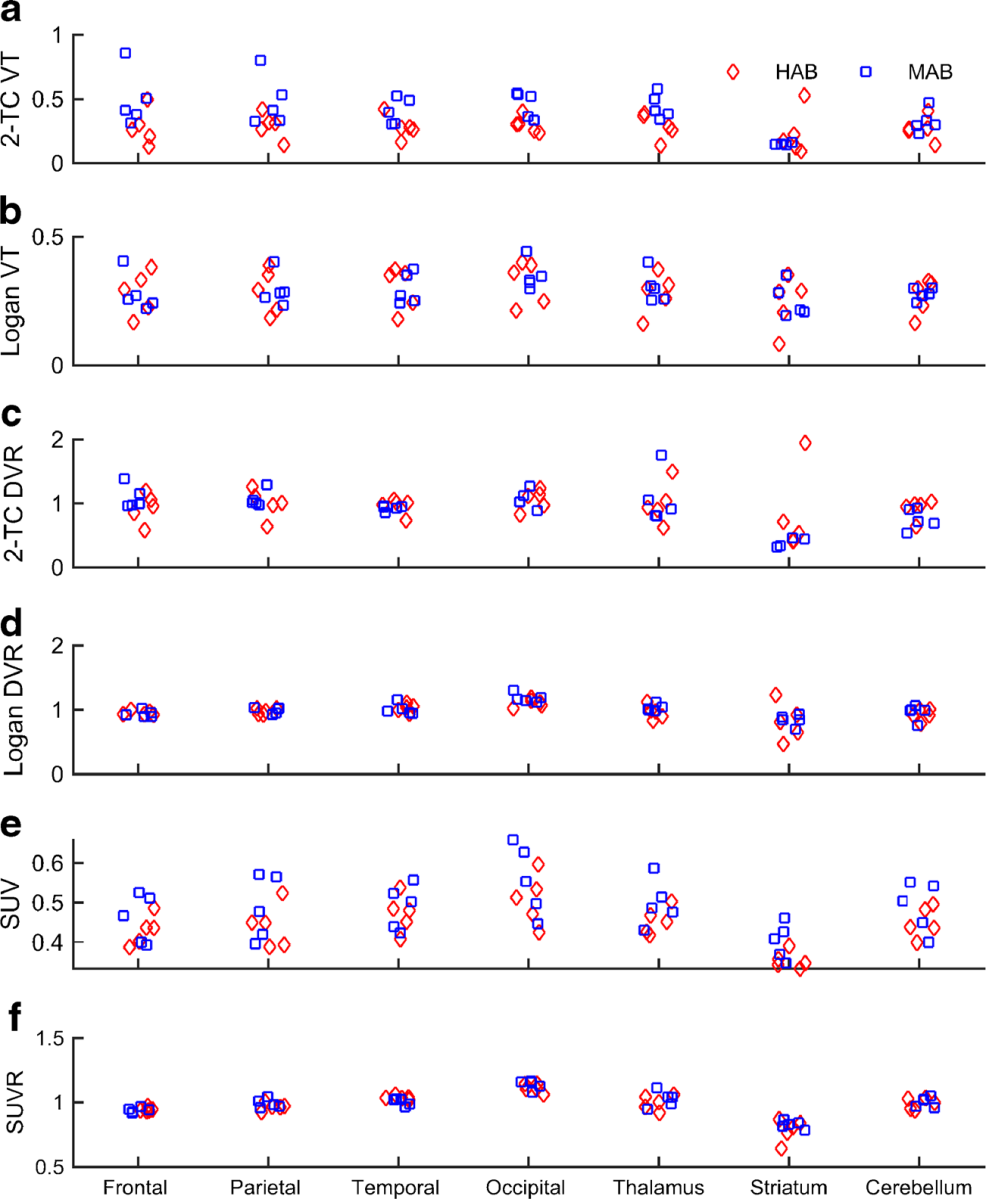
Outcome measures. Six outcome measures for seven regions of interest (*red* high-affinity binders, *blue* mixed-affinity binders). **a** Two-tissue compartment model volume of distribution (*2-TC VT*). **b** Volume of distribution estimated using Logan plot (*Logan VT*). **c** Distribution volume ratio from the two-tissue compartment model using cortical grey matter as a reference region (*2-TC DVR*). **d** Distribution volume ratio estimated using Logan plot and cortical grey matter as a reference region (*Logan DVR*). **e** 60 – 90 min standardized uptake values (**e** *SUV*). **f** 60 – 90 min standardized uptake value ratios (**f** *SUVR*) using cortical grey matter as a reference region

The time stability analysis of the 2TC model demonstrated an increasing *V*_T_ for each successive time window analysed (shown for two ROIs in Fig. 4d). A comparison of six outcome measures for a number of ROIs is shown in Fig. 5. For all six outcome measures, no significant effect of genetic group was found (*p* > 0.186), nor was there any interaction between genetic group and ROI (*p* = 0.468).

## Discussion

Here we describe the characterization and quantification of the novel TSPO tracer [^18^F]GE-180 for the first time in the normal healthy human brain. We generated arterial parent plasma and whole-blood input functions and fitted brain TACs to 1TC and 2TC kinetic models and Logan graphical analysis to generate outcome measures across regions and individuals. The following key outcome measures were generated from the analysis: *K*_1_, *V*_T_, SUV, DVR and SUVR with the cortical grey matter as a pseudo reference region. In addition, we investigated whether the TSPO Ala147Thr polymorphism in the TSPO binding site influenced these outcome measures [14, 22].

There was consistent and stable metabolism of [^18^F]GE-180 parent compound across all individuals. There was no difference in blood or plasma activity between the two genotypes and there were only moderate levels of detectable metabolites in all individuals with 70 % of the intact parent tracer remaining at 90 min. SUV images across all individuals demonstrated low uptake of the tracer in brain tissue with significant tracer activity apparent in the blood compartments of the brain. The low brain uptake could reduce the signal-to-noise ratio for this tracer and might mean that variation in the activity within the blood across the groups could have biased results. In addition, the low uptake might make the tracer more susceptible to showing increased uptake when there is BBB breakdown.

Analysis of the tracer compartment models showed that the reversible 2TC model provided the best overall fit in the majority of cases. There was a small discrepancy in the model fit at the initial sharp peak of the curve, i.e. <5 min of data acquisition. It is possible that this may be due to increased dispersion of [^18^F]GE-180 in the vascular bed, although this is difficult to quantify precisely. In addition, the 1.5-m line that was used from the radial artery to the arterial blood detection machine may have affected the model fit. However, this discrepancy should not significantly affect the estimation of *V*_T_ as this is based on the integral of the impulse response function (i.e. integral/area under the curve of plasma input function) [23]. *K*_1_ could be affected by dispersion but would still remain small after any correction and therefore the interpretation of low brain delivery of this tracer is still valid.

Using the 2TC model, the initial rate constant, *K*_1_, was consistently low, suggesting a low extraction fraction and delivery into brain tissue. Theoretically, there could be a number of reasons for this. First, the low *K*_1_ could be due to low lipophilicity. However, preclinical work has suggested that this tracer is relatively lipophilic (log*D* at pH 7.4 is 2.95), making this unlikely. Second, [^18^F]GE-180 could be a substrate for xenobiotic pumps at the BBB such as the three major ABC transporters, p-glycoprotein (ABCB1), multidrug resistance protein 1 (ABCC1) and mitoxantrone resistance protein (ABCG2), as can be seen with other tracers with low BBB penetration [24]. Third, the low *K*_1_ could be due to increased plasma protein binding, although the relationship here is complex and high plasma protein binding does not always lead to low brain penetration. Most molecules to a greater or lesser extent bind to human serum albumin and some tracers also bind to alpha1-acid glycoprotein [25]. However, in the case of [^18^F]GE-180, the binding affinity to these or other plasma proteins may be considerable. A limitation of this study was that the protein binding of [^18^F]GE-180 was not measured. However, in vitro work suggests that in humans the plasma free fraction is approximately 3 %.

The median volume of distribution of [^18^F]GE-180 using the 2TC model across all subjects and brain regions ranged from 0.16 to 0.38 mL cm^−3^. There was little variability across brain regions. *V*_T_ estimates were lower than those observed for some other second-generation TSPO tracers (e.g. 4.1 ± 1.6 mL cm^−3^ for [^11^C]-PBR-28 in grey matter [26], and 0.72 – 1.06 mL cm^−3^ for [^11^C]PK-11195 [27]).

Our time stability analysis demonstrated that *V*_T_ did not reach a stable level but continuously increased over the 90-min scanning window. This might have led to underestimation of *V*_T_. A scan time of 90 min was originally selected based on preclinical studies and on consideration of what would be acceptable to the individual subjects. However, our results suggest that a longer scanning duration might give a more ‘stable’ *V*_T_ estimate. This ongoing increase in *V*_T_ could have been caused by the accumulation of radiometabolites in the brain. However, only modest levels of metabolites were detected in the blood, and earlier preclinical work demonstrated very low penetration of any metabolite into the brain with 94 % parent at 60 min [28]. Metabolites have not formally been identified, but all those observed in this study were more polar than the parent. It is believed that the two main routes of metabolism are *O*-demethylation and hydroxylation of the aliphatic ring [28]. Other metabolites could be a combination of the two processes or hydroxylation at different sites. The question of accumulation of metabolites in the brain is most relevant when brain uptake is high, which was not the case here. It is also worth noting that these time stability results are consistent with those from other TSPO tracers which also do not achieve a stable estimate within a 2-h scanning window, e.g. [^11^C]PBR28 [19].

No evidence of an effect of TSPO genotype on any of our outcome measures was found. No differences in *K*_1_, *V*_T_, DVR, SUV or SUVR between MABs and HABs were found. This was an unexpected finding, as in vitro work with cold GE-180 displacing [^3^H]PK11195 has shown a binding affinity of 15:1 between HABs and LABs (D. Owen, personal communication). Although we did not acquire PET scans in LABs, we would still have expected to see differences in the outcome measures between HABs and MABs. Our expectation was that MABs would show an intermediate binding relative to that in HABs, i.e. around 50 % of the binding in HABs [13]. The fact that we did not observe such a difference in vivo may have been a consequence of the low uptake seen with this tracer. Genotype effects may be found in other groups, for example older individuals or diseased individuals in whom we may expect microglial TSPO expression to be higher. We did consider the effect that increasing age of the subjects could have had on TSPO binding, as has been shown previously [29], but we did not find any correlations between age and *V*_T_ in any of the ROIs.

In summary, we report the first PET studies of [^18^F]GE-180 in humans. Administration of the tracer was safe and the scan was tolerated well by all subjects. A reversible 2TC model fitted the data well and determined that the tracer has a low first-pass extraction (about 1 %) and low *V*_T_ estimates in our healthy subjects. There was no observable dependency on the rs6971 polymorphism as compared to other second-generation TSPO PET tracers. A low first-pass extraction combined with a tissue signal with a relatively large blood component suggests similarities to [^11^C]PK-11195 in vivo. However, more work with [^18^F]GE-180 in humans would be informative and should include studies in patients with neuroinflammatory conditions to assess signal in the presence of upregulated TSPO, studies in subjects of various ages and a competition study to more clearly delineate specific binding.

## Electronic supplementary material

Below is the link to the electronic supplementary material.

Supplementary Table 1 Subject demographics (*HAB* high affinity TSPO binders, *MAB* mixed affinity TSPO binders) (PDF 52 kb)

Supplementary Figure 1 Parent in plasma for HABs and MABs over time (PNG 95 kb)

Supplementary Figure 2 First 10 min of Fig. 4a, c shown in more detail. **a** Model fits are shown for one-tissue compartment model (reversible 1TC, *dashed line*) and two-tissue compartment model (reversible 2TC, *red line*) against a time–activity curve (*black dots*) for the parietal lobe. **c** Parietal lobe time–activity curve (*solid line*), 5 % whole-blood activity curve (*red line*) and the curve of the difference between the two curves (*dashed line*) highlights the significant contribution of blood signal to the time–activity curve (PNG 234 kb)
