# Supplementary material for: Kinetic analysis of the translocator protein positron emission tomography ligand [18F]GE-180 in the human brain
Source: Eur J Nucl Med Mol Imaging. 2016 Jun 28;43(12):2201–10. doi: 10.1007/s00259-016-3444-z (PMC5047949; doi:10.1007/s00259-016-3444-z)
Supplement: Supplementary file 1 — Subject demographics (HAB high affinity TSPO binders, MAB mixed affinity TSPO binders) (PDF 52 kb) [file 259_2016_3444_MOESM1_ESM.pdf]

Supplementary Table 1. Subject demographics. HAB=high affinity TSPO binders and MAB=mixed affinity TSPO binders

Subject	Genotype	Age (yrs)	Gender	Weight (kg)
1	HAB	47.9	M	97.9
2	HAB	35.5	M	72.1
3	HAB	34.4	M	94.6
4	HAB	48.1	F	69.7
5	HAB	28.4	M	96.6
6	MAB	49.2	M	87.5
7	MAB	39.8	F	70.3
8	MAB	31.0	F	74.2
9	MAB	56.2	M	65.4
10	MAB	39.7	M	90.4
