# Supplementary figures and images for: Kinetic analysis of the translocator protein positron emission tomography ligand [18F]GE-180 in the human brain
Source: Eur J Nucl Med Mol Imaging. 2016 Jun 28;43(12):2201–10. doi: 10.1007/s00259-016-3444-z (PMC5047949; doi:10.1007/s00259-016-3444-z)

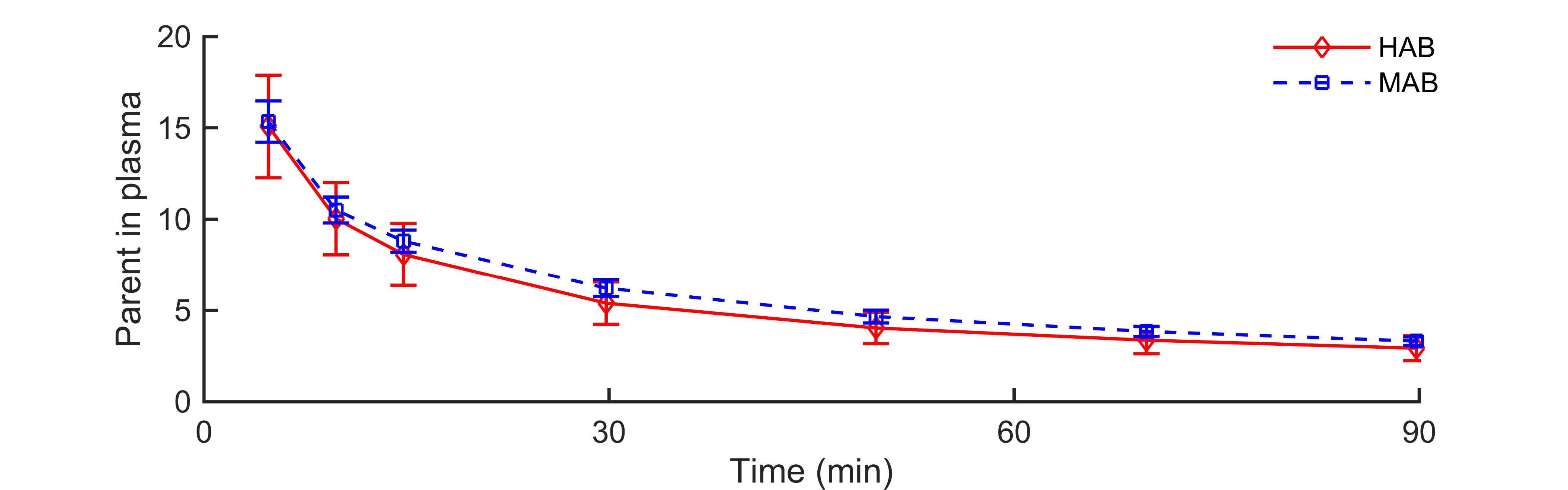

Supplement: Supplementary file 2 — Parent in plasma for HABs and MABs over time (PNG 95 kb) [file 259_2016_3444_MOESM2_ESM.png]

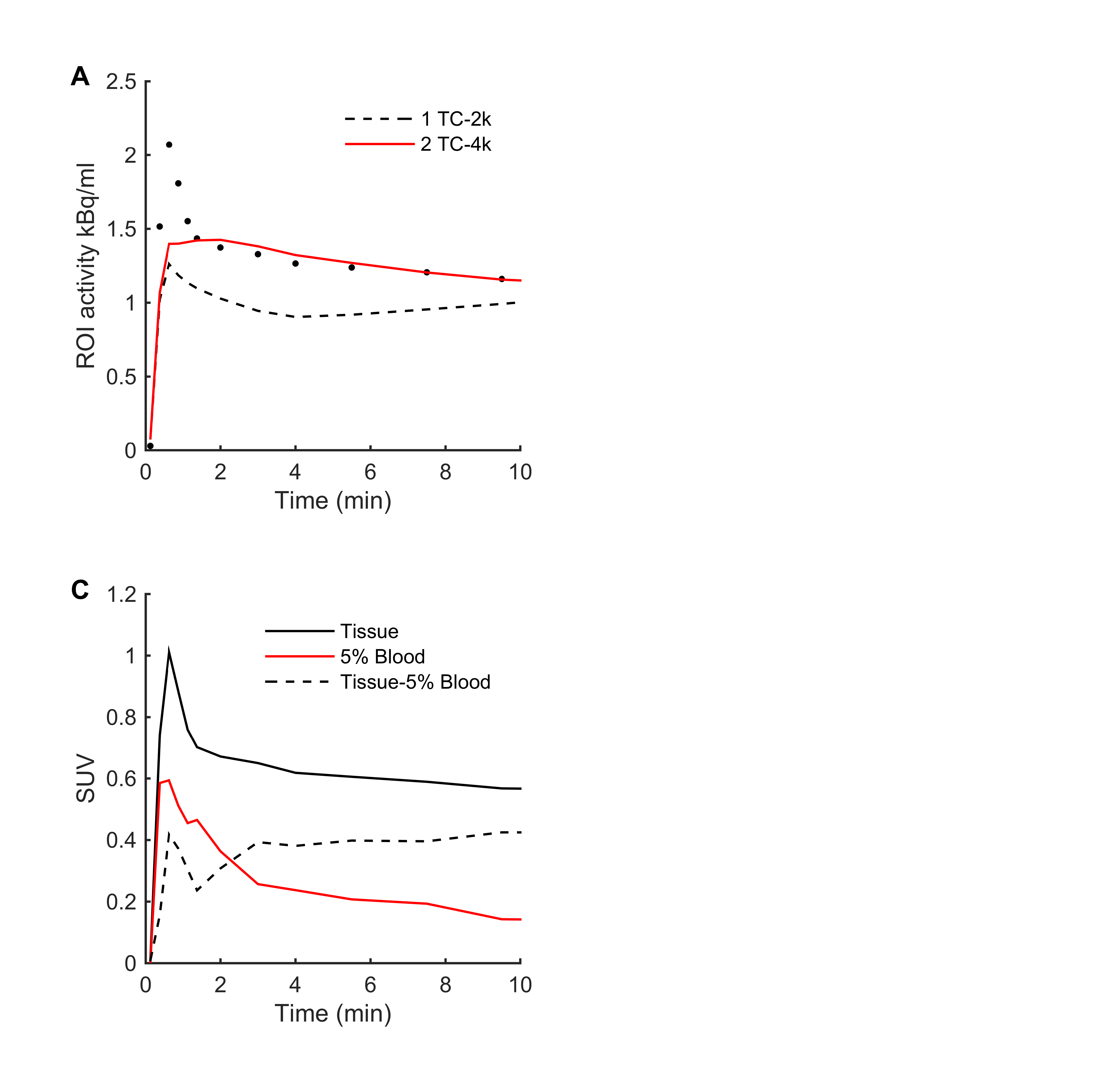

Supplement: Supplementary file 3 — First 10 min of Fig. 4a, c shown in more detail. a Model fits are shown for one-tissue compartment model (reversible 1TC, dashed line) and two-tissue compartment model (reversible 2TC, red line) against a time–activity curve (black dots) for the parietal lobe. c Parietal lobe time–activity curve (solid line), 5 % whole-blood activity curve (red line) and the curve of the difference between the two curves (dashed line) highlights the significant contribution of blood signal to the time–activity curve (PNG 234 kb) [file 259_2016_3444_MOESM3_ESM.png]
